# Joint Effect of Genetic and Lifestyle Risk Factors on Type 2 Diabetes Risk among Chinese Men and Women

**DOI:** 10.1371/journal.pone.0049464

**Published:** 2012-11-21

**Authors:** Raquel Villegas, Ryan Delahanty, Yu-Tang Gao, Jirong Long, Scott M. Williams, Yong-Bing Xiang, Hui Cai, Hong-Lan Li, Frank Hu, Qiuyin Cai, Wei Zheng, Xiao-Ou Shu

**Affiliations:** 1 Department of Medicine, Vanderbilt Epidemiology Center, Vanderbilt University Medical Center, Nashville, Tennessee, United States of America; 2 Department of Epidemiology, Shanghai Cancer Institute, Shanghai, Peoples’ Republic of China; 3 Geisel School of Medicine, Dartmouth College, Hanover, New Hampshire, United States of America; 4 School of Public Health, Harvard University, Boston, Massachusetts, United States of America; Brigham and Women’s Hospital and Harvard Medical School, United States of America

## Abstract

More than 40 genetic susceptibility loci have been reported for type 2 diabetes (T2D). Recently, the combined effect of genetic variants has been investigated by calculating a genetic risk score. We evaluated 36 genome-wide association study (GWAS) identified SNPs in 2,679 T2D cases and 3322 controls in middle-age Han Chinese. Fourteen SNPs were significantly associated with T2D in analysis adjusted for age, sex and BMI. We calculated two genetic risk scores (GRS) (GRS1 with all the 36 SNPs and GRS2 with the 14 SNPs significantly associated with T2D). The odds ratio for T2D with each GRS point (per risk allele) was 1.08 (95% CI: 1.06–1.09) for GRS1 and 1.15 (95% CI: 1.13–1.18) for GRS2. The OR for quintiles were 1.00, 1.26, 1.69, 1.95 and 2.18 (P<0.0001) for GRS1 and 1.00, 1.33, 1.60, 2.03 and 2.80 (P<0.001) for GRS2. Participants in the higher tertile of GRS1 and the higher BMI category had a higher risk of T2D compared to those on the lower tertiles of the GRS1 and of BMI (OR = 11.08; 95% CI: 7.39–16.62). We found similar results when we investigated joint effects between GRS1 and WHR terciles and exercise participation. We finally investigated the joint effect between tertiles of GRSs and a composite high risk score (no exercise participation and high BMI and WHR) on T2D risk. We found that compared to participants with low GRS1 and no high risk factors for T2D, those with high GRS1 and three high risk factors had a higher risk of T2D (OR = 13.06; 95% CI: 8.65–19.72) but the interaction factor was of marginal significance. The association was accentuated when we repeated analysis with the GRS2. In conclusion we found an association between GRS and lifestyle factors, alone and in combination, contributed to the risk of and T2D among middle age Chinese.

## Introduction

Type 2 diabetes (T2D) is a complex disease affecting more than a billion people worldwide. Over the last two decades, China, like many other Asian countries, has experienced a dramatic increase in T2D incidence. Both genetic and environmental factors contribute to the development of T2D. Identifying genetic factors that contribute to T2D and their interactions with environmental factors can lead to prevention and lowering incidence of disease.

Through genome-wide association studies (GWAS), several genetic susceptibility loci have been reported for T2D [1]–[8]. Most of the reported genetic variants have small to moderate effects and account for only a small proportion of the heritability of T2D. Some data is available on the joint effects of common genetic variants on the risk of T2D [9]–[14]. However, most of these studies have not taken into consideration interactions between genetic risk and environmental factors. Looking at the joint effect between genetic risk and lifestyle factors show a large effect size on associations with T2D might help to identify high risk populations for intervention and to elucidate the biology behind the associations between genes and T2D.

In this report, we evaluated the association between reported SNP from genome-wide association study (GWAS) with T2D using data from a GWAS study of T2D of middle age women and from a nested case control study from two population based cohorts of middle age men and women living in urban Shanghai, China and calculated genetic risk scores (GRSs) with T2D susceptibility markers. We also looked at joint associations between genetic risk score tertiles with tertiles of body mass index (BMI), waist to hip ratio (WHR) and exercise participation and with a composite lifestyle high risk score including high BMI, high WHR and no exercise participation.

## Methods

### Ethics Statement

The study protocols were approved by the Institutional Review Boards of Vanderbilt University and the Shanghai Cancer Institute, and all participants provided written, informed consent.

### Study Design and Population

Participants for the present study come from the Shanghai Diabetes GWAS Study and from a nested case control study of T2D identified from two prospective cohort studies the Shanghai Women’s Health Study (SWHS) and the Shanghai Men’s Health Study (SMHS).

#### The Shanghai Diabetes GWAS Study (SDGS)

The SDGS includes genome wide scan data of 1,019 diabetes cases and 1,710 controls. Details of the study design have been described elsewhere [5]. Briefly, diabetes cases in the SDGS included 886 incident T2D cases identified in the Shanghai Women’s Health Study (SWHS), a population-based cohort study of 74,941 women [15] and 133 prevalent T2D cases identified from female controls of the Shanghai Breast Cancer Study (SBCS), a population-based case-control study [16]. The 886 diabetes cases identified from the SWHS all met the following criteria: (1) age ≤65 with a self-reported diabetes diagnosed after study enrollment; (2) used diabetes medication; (3) had fasting glucose level >7 mmol/L at least twice, and (4) donated a blood sample. The 133 diabetes cases identified from the controls of SBCS were women who were diagnosed with T2D and were on diabetes medication or had a blood glucose level >7 mmol/L (measured by study). The 1,710 controls used in this GWAS were shared with a GWAS of breast cancer that was recently completed and was based primarily based on the Shanghai Breast Cancer Study (SBCS). Excluded from the control group are women who (1) had a self-reported history of diabetes; or (2) had a blood glucose level between 5.5 and 7 mmol/L and had HbA1C >6.1% or had no HbA1C data.

The SWHS and the SMHS are both population-based, prospective cohort studies based in urban Shanghai, China. The SWHS began first and recruited 74,941 women aged 40–70 years from 1997 to 2000 The SMHS recruited 61,491 men aged 40–74 years from 2002 to 2006 [17]. The validation sample includes 967 incident T2D cases and 913 controls from the SWHS [15] and 733 male incident T2D cases and 734 controls from the SMHS [18].

#### Anthropometric measurements and exercise participation

Body weight and height were measured in the SWHS, the SMHS and SBCS using identical protocols. All anthropometric measurements including weight, height, and circumferences of waist and hips were taken twice during the in-person interview according to a standard protocol by trained interviewers who were retired medical professionals [19]. From these measurements, the following variables were created: body mass index (BMI), weight in kg divided by the square of height in meters and waist-hip ratio (WHR), waist circumference divided by hip circumference. Physical activity patterns were assessed during the in-person interviews. Regular exercise and sports participation were evaluated for the past 10 years in the SBCS and for the past 5 years in the SWHS and in the SMHS using validated questionnaires [20], [21].

### SNP Selection and Genotyping

The National Human Genome Resource Institute’s (NHGRI) GWAS catalog [Accessed 10/12/2010] [22], was used to identify single nucleotide polymorphisms (SNPs) previously associated with high T2D. We selected 35 SNPs that had been associated with T2D in recent GWAS studies (October 2011) that had a minor allele frequency in Han Chinese (according to HAPMAP CHB group) of at least 5%. The SNPs selected were BCL11A (rs243021), RBMS1, ITGB6 (rs7593730), IRS1 (rs7578326, rs2943641), ADAMTS9 (rs4607103), IGF2BP2 (rs4402960), CDKAL1 (rs7756992, rs10440833), JAZF1 (rs864745), KLF14 (rs972283), TP53INP1 (rs896854), SLC30A8 (rs13266634), CDKN2A, CDKN2B (rs10811661, rs564398), CDC123, CAMK1D (rs12779790), HHEX, IDE (rs5015480, rs1111875), KCNQ1 (rs2237892), KCNJ11 (rs5215), intergenic (rs9300039), MTNR1B (rs1387153), HMGA2 (1531343), TSPAN8, LGRS (rs7961581, rs4760790), C2CD4A, C2CD4B (rs7172432), FTO (rs8050136, rs11642841), SRR (rs391300), HNF1B, TCF2 (rs4430796), DUSP9 (rs5945326), PTPRD (rs17584499), CHCHD9 (rs13292136), CENTD2 (rs1552224), ZFAND6 (rs11634397). In addition, 4 SNPs identified in a meta-analysis of the Asian consortium were also included: KCNK15 (rs3734618), SPRY2 (rs1215468), CMIP (rs12599890) and *FITM2-R3HDML-HNF4A (*rs6017317).

#### Genotyping, for the SDGS study and Imputation

Genotyping was performed using the Affymetrix 6.0 array. The Birdseed v2 algorithm (http://www.broad.mit.edu/mpg/birdsuite/) was used to call genotypes. QC procedures included removal of SNPs with MAFs<0.01, Hardy-Weinberg *P-*values less than 0.00001, and samples with more than 5% missing genotypes. There were 15 SNPs that had been genotyped and the other SNPs that have been reported to be associated with T2D that were not available were imputed using the program MACH (http://www.sph.umich.edu/csg/abecasis/MACH/), using the phased Asian data from HapMap Phase II (release 22) as the reference. Only data with high imputation quality (RSQR>0.3 for MACH) were included in the current analysis.

#### Genotyping for the nested case control study

Genotyping for the 39 SNPs included in the SWHS and SMHS sample set was completed using the iPLEX Sequenom MassArray platform. Included in each 96-well plate as quality control samples were two negative controls, two blinded duplicates and two samples included in the HapMap project. There were 2 SNPs that failed analysis (rs231362 and rs1531343), and one SNP was out of Hardy-Weinberg equilibrium HWE (P<10^−4^); (rs5945326) leaving a total of 36 SNPs for analysis.

#### Data analyses

A total of 74 participants with whom data on four or more genotypes were missing were excluded from the analysis leaving 6,001 participants for the analyses. Demographic and lifestyle parameters were compared between cases controls using Mann Whitney rank sum tests for age, as this variable is not normally distributed, or ANOVA, for other continuous variables. Chi-squared statistics were used to evaluate differences between cases and controls for categorical variables. Single-marker association analyses were carried out to the selected SNP associations with T2D risk. Odds Ratios (ORs) and 95% confidence intervals (CI) were estimated using logistic regression models with adjustment for age, gender and BMI. The association between genotype and T2D risk was evaluated based on an additive genetic model, indexing exposure to risk allele reported in literature. To determine our power to detect previously reported effect sizes across a range of allelic frequencies, we used the program PS, to model T2D as a categorical variable with a fixed sample size under an additive model. [23]. To determine our power to detect an effect, we used independent case-control numbers with a ratio of 1.24, reflecting the control: case ratio (3,322 controls to 2,679 cases). We tested our power to detect association at an alpha of 0.05 (two-sided) under the additive model and used using an uncorrected chi-square test.

#### Genetic risk score

We calculated two risk scores, one with all the SNPs (GRS1) and one with those SNPs that were significantly associated with T2D in the present study (GRS2). We assumed an additive genetic model for each SNP applying a linear weighting of 0, 1 or 2 to genotypes containing 0, 1 or 2 risk alleles respectively. We also calculated weighted GRS by multiplying the GRS by a weighted factor, calculated as = (number of risk alleles/risk alleles-missing genotypes). We checked the Linkage disequilibrium among SNPs in the same loci to see if the r^2^ was over 0.80 before calculating the GRSs and found this not the case.

We evaluated the joint effect of the GRSs and obesity categories according to the WHO recommendations [24], WHR categories (tertiles) and exercise participation (yes/no). We also developed a composite lifestyle high risk score by adding the number of risk factors associated with higher risk of T2D: having high BMI, (>25 kg/m^2^), having a high WHR (>0.85), and no exercise participation. Multiplicative interactions between GRSs and BMI, WHR or exercise participation were examined using the log likelihood ratio test, which compared the model including only the main effect with the model that included both the main effects and the interactive terms. Interaction terms were coded as the product of the genetic and environmental factors under investigation and were considered residual effects after main effects for each SNP. The unknown regression coefficient parameters were estimated using maximum likelihood, conditional on the random variables for genetic, environmental factors, and their products.

All analyses were performed using SAS (version 9.1). All P values presented are based on two-tailed tests. P values presented in this paper were not corrected for multiple testing.

## Results

Associations of selected demographic characteristics and risk factors with T2D are shown in Table 1. As expected, cases and controls differed in regards to age, BMI, WHR, exercise participation and combined high risk factor score. Table 2 presents the association of GWAS-identified, T2D-related SNPs with T2D adjusted for BMI, age and sex in our study population. Fourteen variants in twelve gene regions (*IGF2BP2*, *CDKAL1, KCNK15, TP53INP1, SLC30A8, CDKN2A/CDKN2B, CDC123/CAMK1D, HHEX/IDE, KCNQ1, KCNJ11, SPRY2* and *HNF1B/TCF2)* were significantly associated with T2D. All the SNPs that were significantly associated with T2D in our study (*P*<0.05), had a direction of association consistent with prior GWAS. Further, 23 of 36 SNPs associated with T2D by GWAS showed a consistent direction of association (as indicated by OR) with T2D in our study population (P = 0.066, binomial sign test).

**Table 1:** Characteristics of the study population.

	Controls	Cases	*P* value
**Age (yrs)** Median	53.16	58.57	<.0001
**BMI (mean)**	23.7	26.4	<.0001
**WHR (mean)**	0.83	0.87	<.0001
**Men (%)**	21.46	26.76	<.0001
**BMI categories (%)**			
< = 25	68.4	36.4	<0.001
>25–<30	27.3	49.3	
> = 30	4.3	14.3	
**WHR terciles (%)**			
T1	42.9	18.9	0.001
T2	30.5	32.8	
T3	26.6	48.3	
**PA (%)**			0.07
No exercise	63.6	61.3	
Exercise	36.4	38.7	
**Combined Risk factors**			
**0**	17.0	7.6	0.001
**1**	47.8	26.7	
**2**	25.6	41.7	
**3**	9.6	24.0	

1 P-value for comparison of using Wilcoxon Two-sample Tests. For comparison of categorical variables a χ^2^-square test was used.

**Table 2:** Association of GWAS-Identified T2D-Related SNPs with T2D Risk.

Locus	rsID	Nearby Gene	Effect allele†	Other allele		effect allele freq	OR (95% CI)††		P value	GRS2*
1	rs243021	BCL11A	A		G	0.68	1.03(0.95–1.12)	0.47		
2	rs7593730	RBMS1, ITGB6	C		T	0.83	1.03(0.93–1.15)	0.56		
3	rs7578326	IRS1	A		G	0.85	0.99(0.88–1.12)	0.87		
4	rs2943641	IRS1	C		T	0.93	1.08(0.93–1.26)	0.33		
5	rs4607103	ADAMTS9	C		T	0.63	0.98(0.90–1.06)	0.57		
6	rs4402960	IGF2BP2	T		G	0.25	1.19(1.09–1.30	0.0002		Y
7	rs7756992	CDKAL1	G		A	0.52	1.16(1.07–1.26)	0.0002		Y
8	rs10440833	CDKAL1	A		T	0.40	1.27(1.17–1.38)	<0.0001		Y
9	rs3734618	KCNK15	A		G	0.45	1.10(1.02–1.19)	0.02		Y
10	rs864745	JAZF1	T		C	0.77	0.96(0.88–1.06)	0.44		
11	rs972283	KLF14	G		A	0.72	1.09(1.00–1.19)	0.06		
12	rs896854	TP53INP1	T		C	0.32	1.16(1.06–1.26)	0.0007		Y
13	rs13266634	SLC30A8	C		T	0.59	1.09(1.01–1.19)	0.03		Y
14	rs17584499	PTPRD	T		C	0.10	1.13(0.97–1.32)	0.12		
15	rs564398	CDKN2A, CDKN2B	T		C	0.88	1.06(0.94–1.20)	0.35		
16	rs10811661	CDKN2A,CDKN2B	T		C	0.54	1.20(1.11–1.30)	<0.0001		Y
17	rs13292136	CHCHD9	C		T	0.90	0.99(0.87–1.13)	0.93		
18	rs12779790	CDC123,CAMK1D	G		A	0.17	1.11(1.00–1.24)	0.05		Y
19	rs1111875	HHEX,IDE	C		T	0.30	1.22(1.12–1.33)	<0.0001		Y
20	rs5015480	HHEX,IDE	C		T	0.17	1.34(1.21–1.49)	<0.0001		Y
21	rs2237892	KCNQ1	C		T	0.67	1.20(1.10–1.31)	<0.0001		Y
22	rs5215	KCNJ11	T		C	0.40	1.15(1.06–1.24)	0.0008		Y
23	rs9300039	Intergenic	C		A	0.75	1.07(0.97–1.17)	0.18		
24	rs1552224	CENTD2	A		C	0.92	1.08(0.94–1.25)	0.26		
25	rs1387153	MTNR1B	T		C	0.42	0.99(0.91–1.07	0.72		
26	rs4760790	TSPAN8,LGR5	A		G	0.24	0.99(0.90–1.09)	0.84		
27	rs7961581	TSPAN8,LGR5	C		T	0.21	0.99(0.90–1.09)	0.84		
28	rs1215468	SPRY2	A		G	0.72	1.27(1.16–1.38)	<0.001		Y
29	rs7172432	C2CD4A,C2CD4B	G		A	0.39	0.96(0.88–1.04)	0.27		
30	rs11634397	ZFAND6	G		A	0.09	0.92(0.80–1.05)	0.21		
31	rs8050136	FTO	A		C	0.12	1.08(0.96–1.22)	0.22		
32	rs11642841	FTO	A		C	0.04	1.00(0.81–1.23)	1.00		
33	rs12599890	CMIP	T		C	0.72	0.94(0.87–1.08)	0.14		
34	rs391300	SRR	C		T	0.71	0.99(0.91–1.02)	0.85		
35	rs4430796	HNF1B,TCF2	G		A	0.30	1.18(1.05–1.31)	0.004		Y
36	rs6017317	FITM2-R3HDML-HNF4A	G		T	0.43	1.08(1.00–1.17)	0.06		

T2D increasing risk allele identified in prior GWAS study.

†† ORs and 95% confidence intervals for association with T2D, adjusted for age, sex and BMI.

* SNP is used in the calculation of the GRS2 genetic risk score (see Methods).

According to our power calculations, we only had 88% power to detect significance (P<0.05) for alleles with MAF of 0.26 (the average MAF in this study) and ORs of 1.2 or greater. The average OR for the 14 SNPs for which we detected significance was 1.18.

We calculated two risk scores, GRS1, based on all thirty six SNPs in unique loci, and GRS2, based on the fourteen SNPs significantly associated with T2D. Risk alleles were defined by previously reported T2D GWAS results for all variants included in GRS1 and GRS2. The associations of GRS1 and GRS2 with T2D were evaluated by logistic regression. Both, GRS1, (OR = 1.08; 95% CI: 1.06–1.09) and GRS2 (OR = 1.15; 95% CI: 1.13–1.18), were significantly associated with a higher risk of T2D in analysis adjusted for age, sex and BMI. The OR for quintiles of the GRS1 were 1.00, 1.26, 1.69, 1.95 and 2.18 (P<0.0001) and 1.00, 1.33, 1.60, 2.03 and 2.80 (P<0.001) for GRS2 (Table 3).

**Table 3:** Association between the genetic risk scores and T2D*.

	GRS1		GRS2	
	OR (95% CI)	P value	OR(95% CI)	P value
**Quintiles**				
**Q1**	1.00		1.00	
**Q2**	1.26 (1.07–1.50)		1.33 (1.12–1.58)	
**Q3**	1.69 (1.39–2.05)		1.60 (1.32–1.94)	
**Q4**	1.95 (1.64–2.33)		2.03 (1.69–2.44)	
**Q5**	2.18 (1.81–2.62)		2.80 (2.35–3.35)	
		<0.0001		<0.0001
**Continuous**	1.08 (1.06–1.09)	<0.0001	1.15 (1.13–1.18)	<0.0001

* Adjusted for age, sex and BMI.

The joint effect of BMI categories, WHR categories, and exercise participation, with GRS1 tertiles on T2D was evaluated (Table 4). We found that individuals in the highest categories of GRS1 and categories of BMI (< = or >25 kg/m^2^), WHR (< = or >0.85), and no exercise participation, WHR or who had no exercise participation had the highest ORs for risk of T2D compared with those in the lowest categories or GRS1, low BMI, low WHR or participating in exercise. The P value for interaction was only significant for association between GRS1 and WHR. Participants in the highest tertiles of both GRS1 and higher number of risk factors were more likely to have T2D than controls (OR = 13.06, 95% CI = 8.65–19.72) compared with participants with no risk factor and lower GRS1 category. The P value for the multiplicative interaction factor was of marginal significance.

**Table 4:** Joint Effects of BMI, WHR, exercise participation and combined lifestyle risk factor with GRS1categories on T2D.

	Genetic Risk Score Terciles												
	< = 34			>34–37.21				>37.21					
BMI*	OR	95% CI		OR		95% CI		OR			95% CI		
< = 25	1.00			1.54		1.27–1.87		1.87			1.55–2.26		
25–<30	3.49	2.86–4.26		4.64		3.76–5.73		5.79			4.71–7.13		
> = 30	6.70	4.81–9.32		8.59		5.88–12.55				11.08	7.39–16.62		
*P_interaction_ 0.85*													
**WHR** *	**OR**	**95% CI**		**OR**		**95% CI**		**OR**			**95% CI**		
Low(T1)	1.00			1.37		1.05–1.79		2.05			1.59–2.65		
Medium (T2)	2.37	1.84–3.06		4.01		3.11–5.19		3.83			2.98–4.92		
High (T3)	5.29	4.10–6.83		6.51		4.99–8.49			7.93		6.10–10.32		
*P_interaction_ 0.04*													
**PA** **	**OR**		**95% CI**	**OR**		**95% CI**		**OR**			**95% CI**		
Yes	1.00			1.44		1.15–1.80		1.91			1.53–2.37		
No exercise	1.27		1.04–1.55	1.82		1.48–2.23		2.22			1.81–2.72		
*P_interaction_ 0.79*													
**Combined Factors** *	**OR**		**95% CI**	**OR**		**95% CI**		**OR**			**95% CI**		
none	1.00			1.84		1.19–2.85		2.65				1.75–4.01	
One	1.94		1.34–2.80	2.62		1.82–3.78		3.08				2.14–4.43	
Two	4.88		3.39–7.01	8.03		5.54–11.65				9.53		6.59–13.78	
Three	10.73	7.19–16.02			11.16		7.35–16.93			13.06		8.65–19.72	
*P_interaction_ 0.06*													

* Adjusted for age and sex.

** Adjusted for age, sex and BMI.

+ The log-likelihood ratio test was used to test the interaction effect of GRS with other risk factors on T2D risk.

Table 5 shows joint effects between BMI, WHR, exercise participation and lifestyle combined risk factors with GRS2 (tertiles). We found similar results than those with GRS1 and lifestyle factors, with the joint association between GRS2 and lifestyle factors being stronger although the P values for interaction failed to reach significance.

**Table 5:** Joint Effects of BMI, WHR, exercise participation and combined lifestyle risk factor with GRS2 categories on T2D.

	Genetic Risk Score Terciles							
	< = 9			>9–11.43			>11.43	
BMI*	OR	95% CI		OR	95% CI		OR	95% CI
< = 25	1.00			1.67	1.37–2.05		2.72	2.34–3.30
25–<30	3.88	3.08–4.70		5.37	4.33–6.65		7.76	6.25–9.63
> = 30	7.97	5.58–11.38		8.88	6.19–12.75	14.74		9.84–22.07
*P _interaction_ 0.23*								
**WHR** *	**OR**	**95% CI**		**OR**	**95% CI**		**OR**	**95% CI**
Low(T1)	1.00			1.28	0.97–1.68		3.05	2.35–3.96
Medium (T2)	2.59	1.99–3.38		4.09	3.17–5.29		4.77	3.69–6.17
High (T3)	5.12	3.93–6.67		7.48	5.72–9.78		10.14	7.76–13.24
*P _interaction_ 0.0003*								
**PA** **	**OR**	**95% CI**		**OR**	**95% CI**		**OR**	**95% CI**
Yes	1.00			1.54	1.22–1.93		2.41	1.93–3.02
No exercise	1.25	1.01–1.54		1.86	1.51–2.29		3.04	2.46–3.75
*P _interaction_ 0.96*								
**Combined Factors** *	**OR**	**95% CI**		**OR**	**95% CI**		**OR**	**95% CI**
none	1.00			1.94	1.24–3.05		3.48	2.25–5.39
One	1.93	1.30–2.86		2.58	1.75–3.81		4.57	3.11–6.71
Two	5.21	3.53–7.69		8.91	6.02–13.19		11.70	7.91–17.30
Three	10.65	6.95–16.32	12.63		8.22–19.40		18.13	11.72–28.04
*P _interaction_ 0.10*								

* Adjusted for age and sex.

** Adjusted for age, sex and BMI.

+ The log-likelihood ratio test was used to test the interaction effect of GRS with other risk factors on T2D risk.

## Discussion

In this study we systematically evaluated the combined effects of a genetic risk score and a lifestyle risk score with T2D risk. Participants with a high GRS and high BMI, high WHR, no exercise participation or of a combination of these high risk factors were at increased risk of developing T2D. We only observed and statistically significant P value for interaction between WHR and GRS1.

Some data is available on GRS and T2D risk in European ancestry populations. In a large nested case-control study of 2,809 T2D cases and 3,501 controls from the Health Professionals Follow-up Study and Nurses’ Health Study (all of European ancestry), a. GRS was calculated with 10 SNPs in 9 loci. The odds ratio for T2D with each point of GRS, corresponding to 1 risk allele, was 1.19 (95% CI, 1.14–1.24) for men and 1.16 (CI, 1.12 to 1.20) for women, (12). In a recent study involving an African American population, the trend of increase in risk for T2D with increasing risk allele load was similar to observations in European-derived populations [9]. Some studies have been conducted in Asian populations and have reported similar results. For example the per-allele odds ratio for the development of T2D was 1.12 (95% CI: 1.00–1.25; P = .049) in a Japanese study [10]. In another study conducted in Pakistani population (from the UK and from Pakistan), the GRS using 30 T2D associated SNPs was also associated with a higher risk of T2D [25].

Two studies in China have evaluated associations between genetic risk scores and T2D. In one of the studies, 19 GWAS SNPS were validated using data from two community based studies, a case control study with subjects with T2D, impaired glucose tolerance and normal glucose and data from a prospective study of 734 non diabetic and 67 T2D incident cases [26]. A GRS was calculated using 4 SNPs in the following loci (*CDKAL1, SLC30A8, CDKN2A/CDKN2B* an, *KCNQ1)*. The OR for the case control study (comparing T2D with normal glucose tolerance was 1.28 (1.21–1.35) and that comparing GRS in impaired glucose tolerance with normal glucose tolerance was 1.18 (1.11–1.25). In the prospective study the GRS OR was 1.33 (1.08–1.68). Another cross-sectional study conducted in China of 3,210 participants calculated a GRS using 17 GWAS confirmed SNPs and the odds ratio for T2D (per risk allele) was 1.18 (95% CI 1.12–1.23, p<0.0001) [13], which is similar to what we found in our study and they found a multiplicative interactive effect between BMI with fasting glucose and HbA1c but not with T2D. No joint effect with physical activity and the GRS score was found with T2D or fasting glucose.

In our study no statistically significant interactions were observed between the GRS1 and GRS2 and BMI, exercise participation and combined risk factors. Participants with a high GRS1 and GRS2 and high BMI, no exercise participation, high WHR of a combination of high risk factors were at substantially increased risk of developing T2D compared with those with lower GRS and lower lifestyle risks factors (alone or in combination). These findings suggest the potential value of using genetic markers to identify populations at high risk for T2D for targeted prevention. An interaction between WHR and the GRS1 was found. Our results are in agreement with a recent publication from a prevention trial. In an updated GRS using 34 T2D associated loci, in the Diabetes Prevention Program (DPP) conducted in the U.S. population, the ability of the score to predict diabetes incidence or regression to normal glucose tolerance was tested, and a high GRS was associated with increased risk of developing T2D and lower probability of returning to normal glucose tolerance state in high-risk individuals. They found that a lifestyle intervention attenuates this risk [14].

A strength of this study is that we have included a large number of SNPs identified from NHGRI GWAS catalog. A limitation of the study is that most of the SNPs have been identified from non-Asian populations. Other strengths include that this is a population-based epidemiological study that has comprehensively evaluated GWAS-identified T2D markers in association with T2D risk and interactions with modifiable risk factors of T2D including physical activity levels and a combination of risk factors. The vast majority of our study population is of a single ethnicity, reducing the potential effects of population stratification. The relatively large sample size and the detailed exposure information allowed us to evaluate the joint effect of T2D-related genetic markers and lifestyle risk factors on T2D risk.

Our study has some limitations. The linear models we used for the analysis have reduced power for detecting interactions and this might partially explain the marginal significance of the interactions found in this study. Larger independent studies will be needed to conclusively demonstrate whether the models we have identified here are true examples of effect modification. In addition the SNPs used to construct the GRS do not include many SNPs identified by GWAS studies in Chinese populations. When we did our SNP selection from the GWAS catalogue the majority of the studies had been conducted in white populations. We included SNPs from one study [27] that was published at the time. Other studies have been published since then, including a meta-analysis conducted in East Asian populations [28].

Another limitation is that the study subjects come from different studies and thus it is possible that the heterogeneous study population may have introduced bias in the association tests.

In summary, our study found associations between the GRS and T2D risk. We also found that a joint effect between genetic and modifiable risk factors although the interaction test failed to reach significance. The GRS provides a measure of the combined genetic effect of T2D associated loci.
